# Control and Transferability of Magnetic Interactions in Supramolecular Structures: Trimers of {Cr_7_Ni} Antiferromagnetic Rings

**DOI:** 10.1002/chem.202302360

**Published:** 2023-10-25

**Authors:** Selena J. Lockyer, Deepak Asthana, George F. S. Whitehead, Inigo J. Vitorica‐Yrezabal, Grigore. A. Timco, Eric J. L. McInnes, Richard E. P. Winpenny

**Affiliations:** ^1^ Department of Chemistry The University of Manchester Oxford Road Manchester M13 9PL United Kingdom

**Keywords:** antiferromagnetically coupled, chiral rings, heterometallic rings, multi-spin system, qubit

## Abstract

A synthetic strategy is demonstrated to prepare two distinct trimers of antiferromagnetically coupled {Cr_7_Ni} rings, substantially varying the magnetic interactions between the spin centres. The interactions were studied using multi‐frequency cw EPR: in a trimer linked via non‐covalent H‐bonding interactions no measurable interaction between rings was seen, while in a trimer linked via *iso‐*nicotinate groups isotropic and anisotropic exchange interactions of +0.42 and −0.8 GHz, respectively, were observed. The latter are the same as those for a simpler hetero‐dimer system, showing how the spin‐spin interactions can be built in a predictable and modular manner in these systems.

## Graphical abstract

**Chemical coupling** of antiferromagnetic rings into hetero‐spin supramolecular structures is reported by different routes: control of molecule…molecule magnetic interactions is demonstrated with transferability of parameters from simpler building blocks.

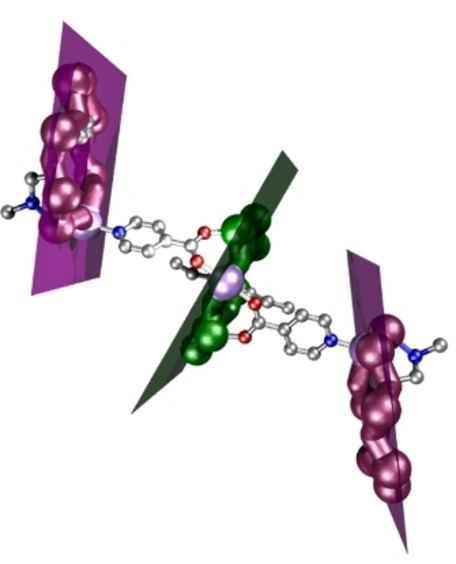

## Introduction

It has been proposed that electron spins within molecules can be used as qubits in quantum information processing.[^1^, ^2^, ^3^] Many groups have focused on the coherence time of the spins, which must be sufficiently long to allow the spins to be manipulated.[^4^, ^5^, ^6^, ^7^, ^8^, ^9^, ^10^, ^11^] However, for the qubits to be useful, they must also interact with other spins in an ensemble. Potentially, chemistry offers advantages here as by control of linking groups it is possible to vary interaction energies over a very wide energy scale. Here we demonstrate this using different links between the same molecular electron spin qubits. This shows the extreme sensitivity of such interactions. We also show that the interactions in the more strongly coupled system are the same as in a hetero‐dimer system with the same connectivity, emphasizing the modularity of the chemistry and physics.

Previously we proposed that {Cr_7_Ni} rings of general formula [(R_2_NH_2_)][Cr_7_NiF_8_(O_2_C^t^Bu)_16_] (**1**) could act as qubits.^[3]^ There are now several classes of heterometallic rings,^[12]^ but the {Cr_7_M} class are appealing because of their tunable magnetic properties and chemical functionalisability.^[13]^ These rings can be bound to other molecules either through functionalizing the secondary ammonium cation which acts as a thread of a rotaxane or *pseudo‐*rotaxane,[^14^, ^15^] or by replacing a carboxylate by a binding group such as *iso*‐nicotinate and binding that to other metals.^[16]^ Recently we have reported a {Cr_7_Ni} ring that uses both methods simultaneously, to bind to a Cu(II) adduct to form a five qubit system with two unique levels of interaction.^[17]^ These {Cr_7_Ni} rings are *green* in colour; from hereon we will use this colour to distinguish them from a related family of rings. EPR spectroscopy of **1** confirms an *S*=1/2 ground state with an average *g*‐value *g_av_* =1.78;^[18]^ the low *g*‐value can be explained from vector coupling^[19]^ considering the strong nearest‐neighbour antiferromagnetic coupling within the ring.

A different ring of formula [Cr_7_NiF_3_(Etglu)(O_2_C^*t*^Bu)_15_(OH_2_)] **2.H_2_O** forms about a quintuply deprotonated N‐ethyl‐D‐glucamine (H_5_Etglu) group, which replaces the R_2_NH_2_ thread and five of the fluorides from the green ring.^[20]^ A terminal water molecule is found on the Ni(II) site and can be easily exchanged with a pyridyl group. Inclusion of *N*‐ethyl‐D‐glucamine results in a chiral {Cr_7_Ni} ring. The change in coordination geometry at many of the chromium sites, and the resultant shift in the Cr(III) dd transitions, leads to a colour change to *purple*.^[21]^ The purple {Cr_7_Ni} also has a well‐isolated *S*=1/2 ground state, but with a slightly higher *g_av_* value of 1.80.^[21]^ We have previously linked purple rings together through di‐ and poly‐imine ligands coordinating at the Ni(II) sites.^[22]^

## Results and Discussion

### Synthesis and structures

In this work we have prepared two distinct three‐ring compounds, each containing two terminal chiral purple {Cr_7_Ni} rings and a central green {Cr_7_Ni} ring. Bond lengths and angles within the rings in the trimers are unchanged from those found in the respective components **1** (R=^n^Pr) and **2.H_2_O** (see Supporting Information).

**Figure 1:**
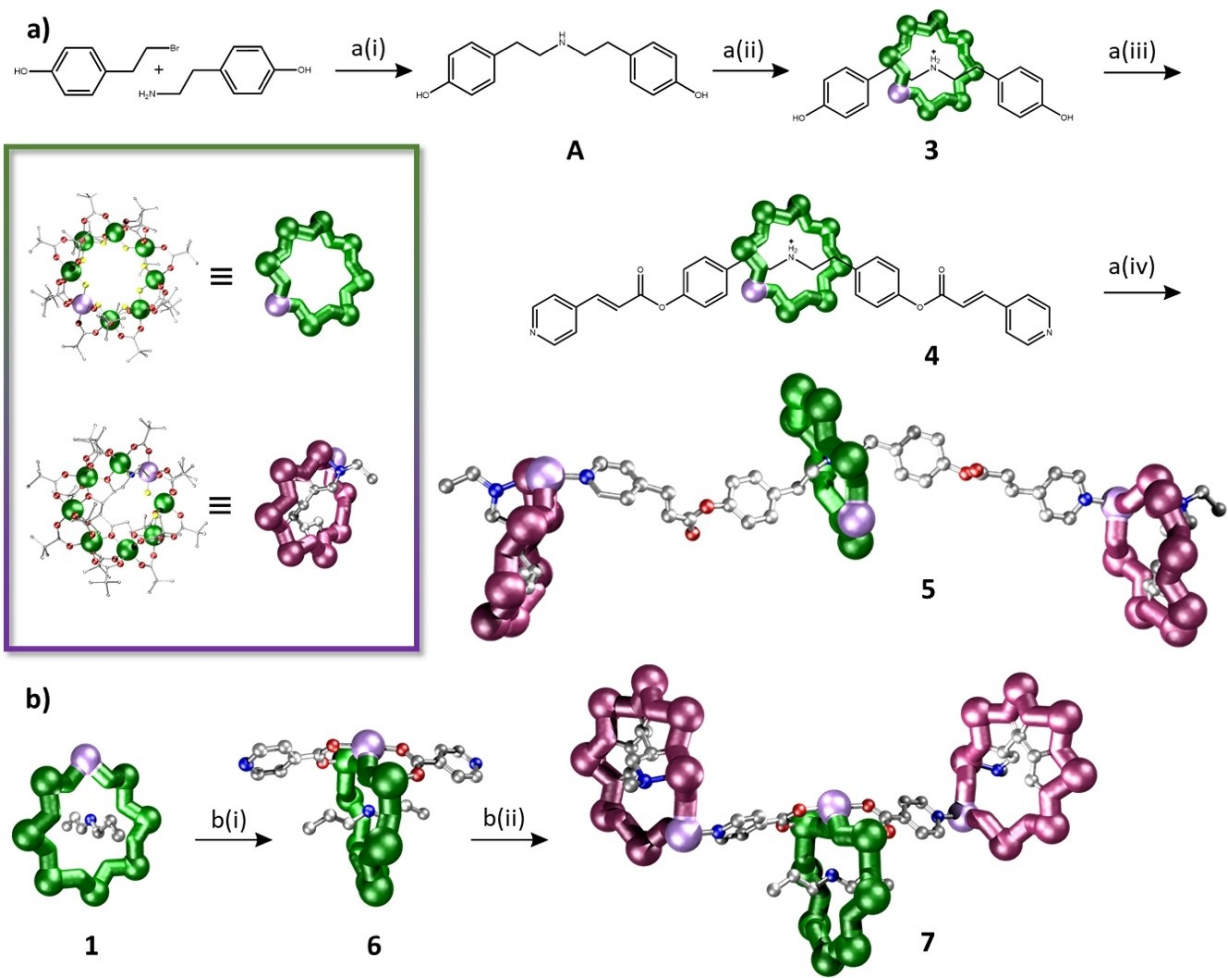
Synthesis for the preparation of multi‐spin systems **5** and **7**. a) Scheme for synthesis of **5**. a(i) DIPEA, DMF, 75 °C, 16 h. a(ii) CrF_3_, xs HO_2_C^t^Bu, nickel carbonate, 160 °C, 24 h. a(iii) DCC, DMAP, THF, 50 °C, 16 h. a(iv) Acetone / pentane. b) Scheme for synthesis of **7**. b(i) *n*PrOH, iso‐nicotinic acid 24 h. b(ii) Acetone / pentane. Insert: Full structure of [Cr_7_NiF_8_(O_2_CBu)_16_]^−^ (top left) and [Cr_7_NiF_3_(Etglu)(O_2_C^*t*^Bu)_15_(OH_2_)] (bottom left); the coloured spheres correspond to the different atom types: Cr (green), Ni (lilac), O (red), N (blue), F (yellow), C (silver). Hydrogens omitted for clarity.

Firstly, we made a rotaxane with a green ring grown about a thread that has a hydroxide group on each end (Figure 1, thread **A**) to form [H**A**][Cr_7_NiF_8_(O_2_C^*t*^Bu)_16_], **3**.^[23]^ Next a Steglich esterification was performed on **3** to add terminal pyridyl groups, using 3‐(4‐pyridinyl)propanoic acid to form [H**B**][Cr_7_NiF_8_(O_2_C^*t*^Bu)_16_], **4** (Figure 1). The terminal pyridyl groups of **4** are both able to bind to purple {Cr_7_Ni} rings in a 1 : 2 reaction with **2.H_2_O** in acetone to form [(**4**){**2**}_2_], **5**. Crystals of **5** were grown from acetone/pentane. The pyridyl groups of the thread bind to the purple {Cr_7_Ni} rings at the Ni(II) site, displacing the H_2_O molecules (Figures 1a, S3).

**Figure 2:**
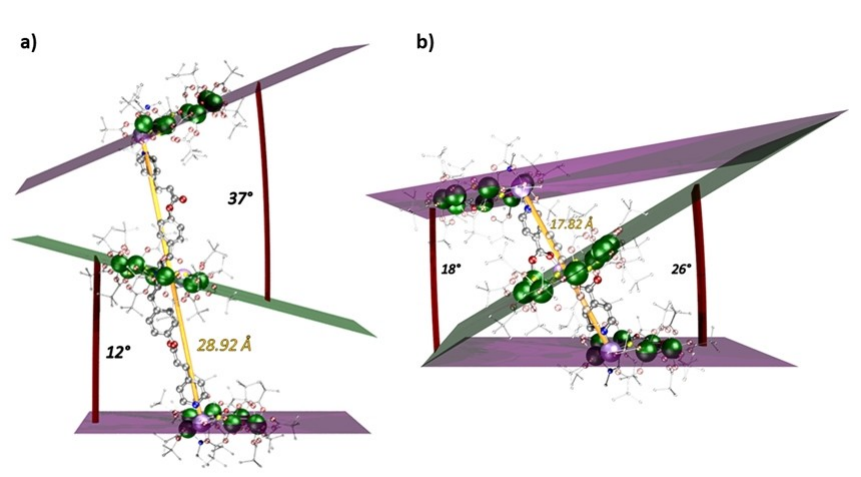
The structures in the crystal of (a) **5** and (b) **7**, including the angles and distances between the {Cr_7_Ni} units. Colour scheme: Cr, green; Ni, lilac; O, red; F, yellow; N. blue; C, grey. H‐atoms excluded for clarity. Mean plane of purple rings shown in purple; mean plane of green rings shown in green. Dihedral angles for each green and purple ring, shown by red arc. Distance between localised Ni(II) atoms in purples ring, depicted by yellow rod.

The structure of **5** is asymmetric with different distances (16.72 and 15.78(1) Å) between the centroid of the green {Cr_7_Ni} ring and the Ni(II) ions in each purple {Cr_7_Ni} ring. The plane of the green {Cr_7_Ni} ring is offset to the planes of the purple {Cr_7_Ni} rings by 12.2 and 37.4°, respectively. The angle between the planes of the two purple {Cr_7_Ni} rings is 26.3° (Figures 2a, S7).

Our second approach is *via* reaction of **1** with *iso*‐nicotinic acid (HO_2_C‐py) to produce [^n^Pr_2_NH_2_][Cr_7_NiF_8_(O_2_C^*t*^Bu)_14_(O_2_C‐py)_2_] **6**, in which two of the pivalates attached to the Ni(II) site are replaced by *iso*‐nicotinate. The pyridyl groups are *trans* to one another and perpendicular to the plane of the ring (Figures 1b, S5). Reaction of **6** with **2.H_2_O** in a 1 : 2 reaction gives [(**6**){**2**}_2_], **7**. Crystals of **7** were grown from acetone/pentane. The distances between the localised Ni(II) ions in the green {Cr_7_Ni} ring and the two purple {Cr_7_Ni} rings are 8.95(1) and 9.00(1) Å. The angles between the plane of the green {Cr_7_Ni} ring and those of the purple {Cr_7_Ni} rings are 26.31 and 17.59° (Figures 2b, S8, S9). The angle between the two purple {Cr_7_Ni} ring planes is 8.37°.

Both **5** and **7** contain a central green {Cr_7_Ni} ring and two terminal purple {Cr_7_Ni} rings but with different connectivity of the spin systems. In **5** there is no direct covalent bond pathway between the green and purple rings while in **7** the substitution connects the green {Cr_7_Ni} ring to each purple {Cr_7_Ni} ring via a short *iso*‐nicotinate linker.

### EPR Spectroscopy

Continuous wave (cw) Q‐band EPR (ca. 34 GHz) spectroscopy measurements were performed on compounds **4**, **5** and **7** as powder samples, and for **7** as a 1 mM solution in dry CH_2_Cl_2_:toluene (1 : 1), at 5 K. CW K‐ and X‐band (ca. 23 GHz and 9.5 GHz, respectively) measurements were also performed on **7**. All spectra were simulated using EasySpin.^[24]^

The spectrum of the [2]rotaxane **4** shows an anisotropic resonance at *ca. g*=1.8 due to the near‐axial *S*=1/2 ground state of the green {Cr_7_Ni} ring (Figure S7; $g_{⊥,∥}^{g}=1.78,\;1.74$ ).^[18]^ Spectra of **2.H_2_O** have been reported,^[21]^ and have better resolution of *g* ($g_{⊥,∥}^{g}=1.84,\;1.78$ ) than for **4**. In the hetero‐trimer **5**, there are two resonances, with a shoulder on the higher field resonance: this can be simulated using the Hamiltonian in Equation (1) which simply adds spectra of purple {Cr_7_Ni} and green {Cr_7_Ni} in a 2 : 1 ratio.

(1) $${\hat{H}}_{1}=\;\sum_{i=1,2}\mu_{B}{\hat{S}}^{p,i}\cdot g^{p,i}\cdot B+\mu_{B}{\hat{S}}^{g}\cdot g^{g}\cdot B\;$$

**Figure 3:**
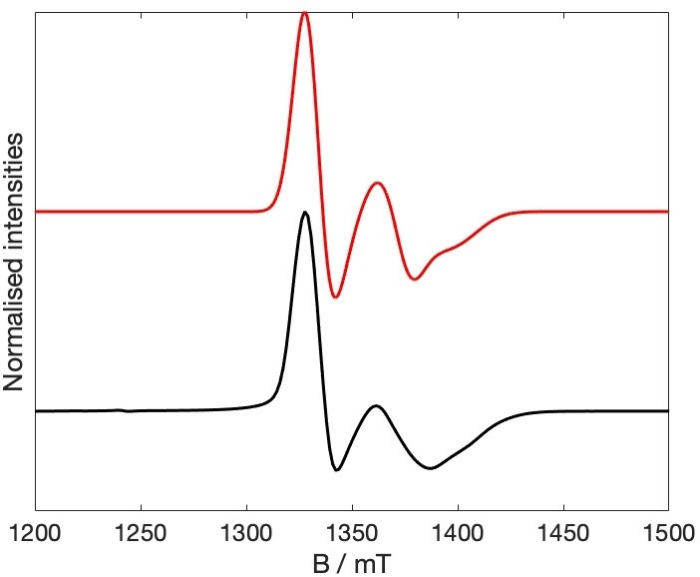
Q‐band EPR spectrum at 5 K for **5** as a powder (black), and simulation (red). Experimental frequency 34.0047 GHz.

In Equation (1), Ŝ^*p,g*^ are the spin operators, and **g** ^*p,g*^ the *g*‐tensors, for the purple (*p*) and green (*g*) {Cr_7_Ni} (*μ*_B_ is the Bohr magneton, **B** is the applied magnetic field). Good agreement is found with $g_{⊥,∥}^{g}$ =1.775, 1.735 and $g_{⊥,∥}^{p}$ =1.825, 1.755, close to those of **4** and **2**, and where ⊥,∥ refer to the local **g**‐frames where the unique axis is perpendicular to the {Cr_7_Ni} planes.[^18^, ^21^] Hence, any interactions between green and purple rings is considerably smaller than the experimental linewidths. The dominant low‐field feature is due to $g_{⊥}^{p}$ ; the high‐field features are due to overlapping $g_{⊥,∥}^{g}$ and $g_{∥}^{p}$ (Figures 3, S10).

**Figure 4:**
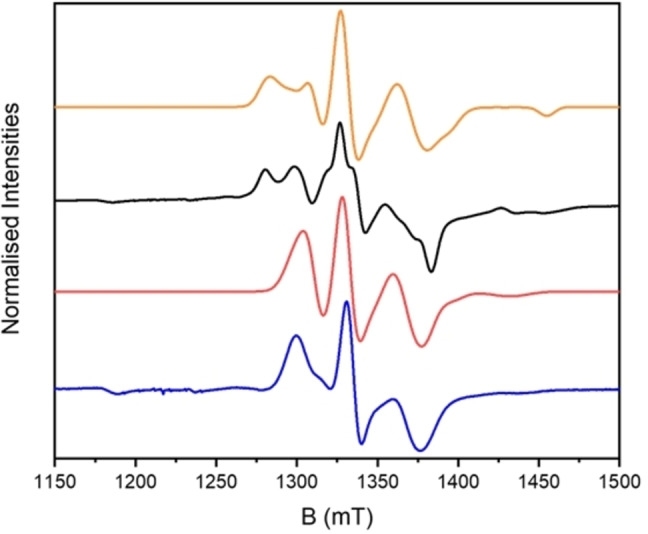
Q‐band EPR spectra for **7** at 5 K, measured as a solution (blue) and solid (black). Calculated spectra for solution (red) and powder (orange) with model as described in the text. Experimental frequency 34.0972 GHz.

In contrast, the EPR spectra of **7** show significant interactions between the three spins, as they do not resemble a simple superposition of those of the components. The solution and solid‐state spectra of **7** are similar, showing three main transitions, however those in the solid state show additional splitting in the outer features (at *ca*. 1300 and 1380 mT at Q‐band, Figures 4, S11).

We began by modelling the simpler solution spectra. We have previously reported a compound closely related to **7**, formed from reaction of the *mono*‐substituted green {Cr_7_Ni}, [^n^Pr_2_NH_2_][Cr_7_NiF_8_(O_2_C^*t*^Bu)_15_(O_2_C‐py)], with **2** giving a purple‐green {Cr_7_Ni} hetero*dimer* linked *via iso*‐nicotinate.^[25]^ Micro‐SQUID magnetisation (at 40 mK, showing a step at ca. 0.16 T) and cw Q‐band EPR measurements (which had the form of a spin triplet with zero‐field splitting, ZFS) were consistent with an antiferromagnetic interaction between the rings. The data could be modelled with a simple Hamiltonian for two interacting *S*=1/2 centres:

(2) $${\hat{H}}_{2}=\;\mu_{B}{\hat{S}}^{p}\cdot g^{p}\cdot B+\mu_{B}{\hat{S}}^{g}\cdot g^{g}\cdot B+J{\hat{S}}^{g}\cdot {\hat{S}}^{p}+{\hat{S}}^{g}\cdot D^{ex}\cdot {\hat{S}}^{p}$$

In Equation (2), *J* is the isotropic exchange interaction and **D** ^ex^ is an anisotropic exchange tensor which we assume to have the traceless and axial form [+1+1−2]*D* ^ex^. This gave *J*=+0.14 cm^−1^ (+4.2 GHz) and *D* ^ex^=−0.03 cm^−1^ (−0.9 GHz) in this model. Given that **7** contains the same building blocks (barring two *vs*. one substituted carboxylates on the green ring) and the same ring‐ring pathways, we attempted to simulate EPR spectra for **7** with a similar Hamiltonian, but with the addition of a second purple ring and with the same parameters:

(3) $${\hat{H}}_{3}=\;{\hat{H}}_{1}+\sum_{i=1,2}\left(+J{\hat{S}}^{g}\cdot {\hat{S}}^{p,i}+{\hat{S}}^{g}\cdot D^{ex}\cdot {\hat{S}}^{p,i}\right)$$

Very good agreement is found using Equation (3) with these fixed parameters. A minor adjustment to *D* ^ex^ to −0.80 GHz gives a slightly better match across Q‐, K‐ and X‐band (Figures 4, S12). The calculations used *g*‐values for **2** ($g_{⊥,∥}^{p}$ =1.845, 1.785) and **6** $(g_{⊥,∥}^{g}$ =1.785, 1.745).[^18^, ^21^] We know from previous studies that the unique axis of **g** ^*g*^ is perpendicular to the {Cr_7_Ni} plane,^[18]^ and we assume the same is true for **g** ^*p*^. Introduction of moderate rotations of the **g** or **D** ^ex^ matrices (consistent with the crystal structure) make minimal differences to calculated spectra. Hence, we have taken **g** ^*p*^, **g** ^*g*^ and **D^ex^** to all be coincident. Slightly better agreements again are found with a gaussian distribution of *D* ^ex^ parameters (FWHM of 40 % about −0.80 GHz, Figure S13).

Using average *g* ^*g*^ and *g* ^*p*^ values of 1.77 and 1.81, respectively, the difference in Zeeman frequency is considerable smaller than *J* even at Q‐band fields (Δ*ν*=0.76 GHz for *B*_0_=1.35 T), hence the spin system can be discussed in the strongly coupled description. In the isotropic limit, the zero‐field eigenfunctions can then be written as |Ŝ^pp^,Ŝ>, where Ŝ^pp^=Ŝ^p,1^+Ŝ^p,2^ and the total spin Ŝ=Ŝ^pp^+Ŝ^g^, and are two doublets (|0,1/2> and |1,1/2>, with eigenvalues 0 and −*J*, respectively) and a quartet (|1,3/2>, at +*J*/2). Hence, the zero‐field ground state of **7** is the |1,1/2> doublet with |0,1/2> at +4.2 GHz and |1,3/2> at +6.3 GHz higher energy.

**Figure 5:**
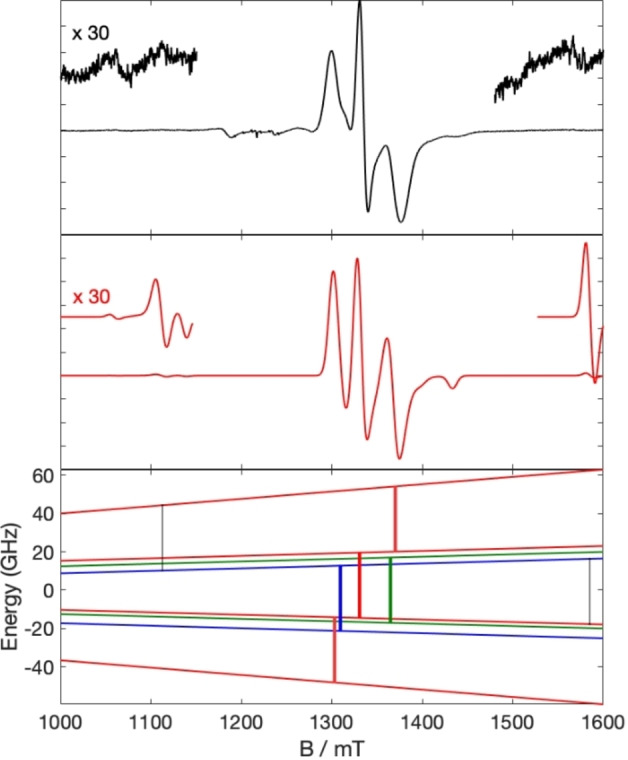
Top: experimental Q‐band spectrum of **7** in solution at 5 K. Middle: Calculated spectrum with the model as in Figure S12 (without *D* ^ex^ strain effects). Bottom: Zeeman diagram for *B*_0_ along the molecular x‐axis, with Q‐band EPR transitions shown. Red, blue and green levels belong to the |Ŝ^*pp*^,Ŝ>=|1, 3/2>, |1, 1/2> and |0, 1/2> total spin states, respectively. Transitions within each state are shown in the respective colour. The shaded transitions at *ca*. 1110 and 1560 mT are “forbidden” Δ*m*=1 transitions between the quartet and the lower energy doublet states (|*S*,*m*>=|1/2,+1/2> to |3/2,+3/2> and |3/2,–1/2> to |1/2,+1/2>, respectively).

The anisotropic exchange terms lead to a ZFS of the quartet state (equivalent to an axial ZFS parameter *D*=+1.54 GHz in a spin Hamiltonian for an isolated *S*=3/2). The EPR spectra are dominated by the quartet (giving the three main features at each frequency; Figure 5) because, even at X‐band, the *m*=−3/2 sublevel is the lowest energy state due to the Zeeman interaction in the applied magnetic fields. The transitions within the two *S*=1/2 states are either obscured by the quartet or appear as shoulders (for example, see Figure S13).

In some of the EPR spectra there are very weak features in the wings (Figure 5). Calculations show these to arise from formally forbidden (Δ*S*=1, Δ*m*=1) transitions between the lower energy doublet (|1,1/2>) and the quartet state: in Hamiltonian [3] these become weakly allowed as a function of the state mixing induced by the anisotropic exchange. The positions of these transitions are sensitive to |*J*|, providing a good handle on this parameter.

The extra structure in the spectra from powder samples suggest a lower symmetry. As noted above, this structure is not reproduced by introducing rotations between **g** ^p^, **g** ^g^ and **D** ^ex^ that would be consistent with the crystal structures. We found that the extra structure can be introduced reasonably by a rhombicity in **D** ^ex^, with trial‐and‐error giving a best agreement with a matrix of the form [+1.25 +0.75 −2]*D* ^ex^ (restricting **D** ^ex^ to being traceless), and a slightly increased *D* ^ex^=−1.1 GHz (Figures 4 and S14,S15). We have shown previously for a series of purple‐purple {Cr_7_Ni} homo‐dimers, where a diimine bridges between the Ni^II^ ions, that that the magnitude of the ZFS within the coupled triplet is a function of the *local* ZFS of the Ni^II^ ions and the Ni…Ni exchange interaction in a spin Hamiltonian based on all 16 metal ions.^[26]^ For the purple‐green systems, bridged between the a Cr−Ni edge on the green ring and the Ni site on purple, it is a function of the local ZFS and the inter‐ring Cr…Ni and Ni…Ni exchange.^[25]^ For **7**, It is possible that the symmetry of the quartet ZFS could change because the relative orientations of the interacting ions (and hence their local ZFSs) relaxes between solid and solution state. However, it is possible that other parameterizations of this extra structure in the solid‐state EPR spectra of **7** are possible.

## Conclusions

We have coupled three spin clusters in two different chemical ways, and demonstrated very different magnetic coupling regimes depending on these pathways. In one, based on a supramolecular rotaxane structure, the exchange interactions between the rings are much smaller than the experimental linewidth of cw EPR spectra. This puts an upper limit of the isotropic exchange of *ca*. 200 MHz. In practice it may be much smaller; we have measured interactions of <10 MHz for [3]rotaxanes based on green {Cr_7_Ni} rings at similar distances.^[27]^ In the other structure, where the three components are linked covalently via coordinate bonds we can measure the interaction by cw EPR as +4.2 GHz, with an anisotropic interaction of −800 MHz. The linkage in this three‐spin system is identical to that in a related two‐spin system, illustrating how complex multi‐component interactions can be built up in a modular and predictable manner.

## Experimental Section

General methods are described in the Supporting Information.

Synthesis of **4**: Compound **3** (0.6 mmol),^[23]^ 3‐(4‐pyridinyl)propanoic acid (3.0 mmol), N,N′‐dicyclohexylcarbodiimide (3.15 mmol) and 4‐dimethylaminopyridine (4.1 mmol) in dry THF (70 mL) were stirred at 50 °C for 16 h in a 250 mL round bottomed flask under N_2_ flow. The solvent was evaporated, and the product isolated by column chromatography (CH_2_Cl_2_/EtOAc). Yield: 36 %. ESI MS m/z (C_112_H_178_Cr_7_F_8_N_3_NiO_36_, M.W.=2717.29 g mol^−1^): 2739.7 [M+Na]^+^. Elemental Analysis: Calculated (found) for C_112_H_178_Cr_7_F_8_N_3_NiO_36_: C, 49.51 (49.80); H, 6.60 (6.64); N, 1.55 (1.75); Cr, 13.39 (12.60); Ni, 2.16 (2.15).

Synthesis of **5**: Compounds **2** (0.1 mmol)^[20]^ and **3** (0.2 mmol) were dissolved in warm acetone (100 mL) and stirred for 5 minutes. The precipitate was filtered, washed with acetone (100 mL), dissolved in pentane (25 mL) and filtered again. Acetone (10 mL) was added and the solution left undisturbed. Needle‐like crystals suitable for single crystal X‐ray analysis were grown within two weeks. Yield: 17 %. Elemental Analysis: Calculated (Found) for C_278_H_476_Cr_21_F_14_N_5_Ni_3_O_106_: C, 46.90 (47.88); H, 6.74 (6.99); N, 0.98 (0.93); Cr, 15.34 (13.35); Ni, 2.47 (2.28).

Synthesis of **7**: Compounds **6** (0.08 mmol)[^28^, ^29^] and **3** (0.17 mmol) were dissolved in warm acetone for 5 minutes. The mixture was filtered and the precipitate dissolved in pentane (50 mL). To the pentane solution, acetone (100 mL) was added and left undisturbed. Needle‐like crystals suitable for single crystal X‐ray analysis were grown within one week. Yield: 23 %. Elemental Analysis: Calculated (found) for C_254_H_448_Cr_21_F_14_N_5_Ni_3_O_102_: C, 45.28 (45.13); H, 6.70 (6.66); N, 1.04 (1.13); Cr, 16.20 (15.43); Ni, 2.61 (2.67).

## Supporting Information

All data further supporting this study is provided in the Supporting Information (SI) file accompanying this paper. Deposition Numbers 2283570, 2283571 and 2058846 contain the supplementary crystallographic data for this paper.^[30]^ The authors have cited additional references within the Supporting Information.[^31^, ^32^] The data that support the findings of this study are available from the corresponding author upon reasonable request.

## Conflict of interest

The authors declare no conflict of interest.

1

## Supporting information

As a service to our authors and readers, this journal provides supporting information supplied by the authors. Such materials are peer reviewed and may be re‐organized for online delivery, but are not copy‐edited or typeset. Technical support issues arising from supporting information (other than missing files) should be addressed to the authors.

Supporting Information

## Data Availability

The data that support the findings of this study are available from the corresponding author upon reasonable request.
