# Supplementary material for: Control and Transferability of Magnetic Interactions in Supramolecular Structures: Trimers of {Cr7Ni} Antiferromagnetic Rings
Source: Chemistry. 2023 Oct 25;29(69):e202302360. doi: 10.1002/chem.202302360 (PMC10947047; doi:10.1002/chem.202302360)
Supplement: Supplementary file 1 — Supporting Information [file CHEM-29-0-s001.pdf]

Chemistry–A European Journal

Supporting Information

Control and Transferability of Magnetic Interactions in Supramolecular Structures: Trimers of $\{Cr_7Ni\}$ Antiferromagnetic Rings

Selena J. Lockyer, Deepak Asthana, George F. S. Whitehead, Inigo J. Vitorica-Yrezabal, Grigore. A. Timco, Eric J. L. McInnes,* and Richard E. P. Winpenny*

Contents

1. Synthesis ***S2***

1.1 Synthesis of [$HOC_6H_4(CH_2)_2NH(CH_2)_2C_6H_4OH$] **A** S2

1.2 Synthesis of [2]rotaxane [($Cr_7NiF_8(O_2C^tBu)_{16}$}H**A**] **3** S3

1.3 Dual Esterification of [2]rotaxane [($Cr_7NiF_8(O_2C^tBu)_{16}$}H**B**] **4** S3

1.4 Synthesis of [Cr_7NiF_3(Etglu)($O_2C^tBu)_{15}(OH_2)$] **2.H$_2$O** S4

1.5 Synthesis of [(**2**){Cr_7NiF_3(Etglu)($O_2C^tBu)_{15}$}$_2$] **5** S4

1.6 Synthesis of [$(CH_3CH_2CH_2)_2NH_2$][$Cr_7NiF_8(O_2C^tBu)_{16}$] **1** S4

1.7 Synthesis of [$(CH_3CH_2CH_2)_2NH_2$][$Cr_7NiF_8(O_2C^tBu)_{14}(C_6H_5NO_2)_2$] **6** S4

1.8 Synthesis of [**6**(Cr_7NiF_3(Etglu)($O_2C^tBu)_{14})_2$] **7** S4

2. Thermogravimetric analysis (TGA) ***S5***

3. Crystallography ***S7***

3.1 Crystallographic Figures S7

3.2 Crystallographic Information S9

3.2 Crystallography Data: **Table 1** S10

4. Electron Paramagnetic Resonance (EPR) Spectroscopy ***S12***

1. Synthesis

General: All starting reagents and materials used were sourced from Sigma-Aldrich and/or Alfa. Unless stated otherwise, no further purification was required for all reagents and solvents. The syntheses of the hybrid organic-inorganic rotaxanes were carried out in Erlenmeyer Teflon® FEP flasks supplied by Fisher. Column chromatography was performed using either 40-63 µm silica from Sigma-Aldrich or a Grace Reverelis ® X2 Autocolumn with Grace Reverelis ® NP cartridges. Chemical shifts are reported in parts per million (ppm) from low to high frequency and referenced to the residual solvent resonance. ESI mass spectrometry and microanalysis were carried out by the services at The University of Manchester.

1.1 Synthesis of [$HOC_6H_4(CH_2)_2NH(CH_2)_2C_6H_4OH$] **A**:[23]

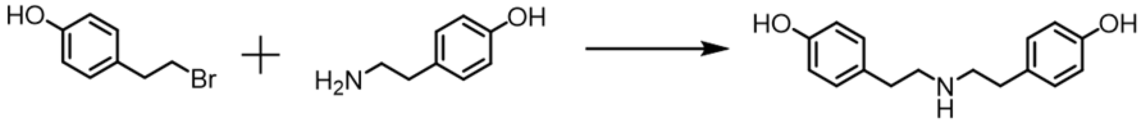

2-(4-Hydroxyphenyl)-1-bromoethane (2.0 g, 9.95 mmol) and 2-(4-Hydroxyphenyl)ethylamine (6.0 g, 43.7 mmol) were added to a solution of 5.2 mL of *N*-Ethyldiisopropylamine (DIPEA) in 45 mL of dry DMF solvent. The reaction mixture was heated at 75 °C for 16h, then cooled to room temperature. The product was extracted with ethyl acetate (EtOAc)/Water. The organic layer was washed with brine and dried over $MgSO_4$. The solvent was evaporated to afford the crude product. The solid residue was dissolved in hot methanol (MeOH) followed by addition of 20 mL water. The crystalline product was filtered with repeated washing using chloroform to get produce the pure product. Yield: 1.8 g 76%. ESI MS m/z ($C_{16}H_{19}NO_2$, M.W. = 257.17 g mol^{-1}): 258 $[M+H]^+$, 280 $[M+Na]^+$. ^1H NMR (400 MHz, DMSO-d_6, 300 K): δ = 9.15 (2H, Ph-O*H*), 6.95 (4H, Ar), 6.64 (4H, Ar), 2.67 (4H, Ph-CH_2), 2.55 (4H, Ph-CH_2CH_2), 1.43 (1H, N*H*). Elemental Analysis: Calculated (found) for $C_{16}H_{19}NO_2$: C, 74.68 (74.03); H, 7.44 (7.45); N, 5.44 (5.45).

1.2 Synthesis of [2]rotaxane $[(Cr_7NiF_8(O_2C^tBu)_{16}\}H\mathbf{A}]$ **3**:[23]

Pivalic acid (45 g), **A** (2.5 g), $CrF_3 \cdot 4H_2O$ (6.0 g) and $[2NiCO_3 \cdot 3Ni(OH)_2 \cdot 4H_2O]$ (1.0 g), were added to a Teflon flask and heated at 140 °C for 24 hrs. The reaction mixture was then cooled to room temperature and filtered with washing from chloroform. The crude product was purified by column chromatography (Silica, $CHCl_3$/EtOAc). Yield: 4.29 g 18%. ESI MS m/z ($C_{96}H_{164}Cr_7F_8NNiO_{34}$, M.W = 2450.9 g mol^{-1}): 2473 $[M+Na]^+$. Elemental Analysis: Calculated (found) for $C_{96}H_{164}Cr_7F_8NNiO_{34}$: C, 47.04 (47.35); H, 6.74 (6.84); N, 0.57 (0.65); Cr, 14.85 (13.78); Ni, 2.39 (2.32).

1.3 Dual Esterification of [2]rotaxane $[(Cr_7NiF_8(O_2C^{t}Bu)_{16}\}H\mathbf{B}]$ **4**:

B: $py(CH_2)_2CO_2C_6H_4(CH_2)_2NH(CH_2)_2C_6H_4CO_2(CH_2)_2py$

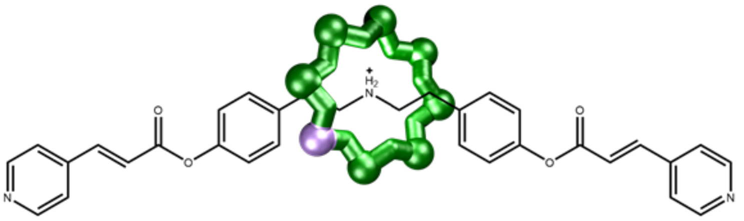

To a 250 mL round bottomed flask under N_2 flow, **3** (1.5 g, 0.6 mmol), was reacted with 3-(4-pyridinyl)propanoic acid (0.46 g, 3.0 mmol), N,N′-Dicyclohexylcarbodiimide (DCC) (0.65 g, 3.15 mmol) and 4-Dimethylaminopyridine (DMAP) (0.50 g, 4.1 mmol) in 70 mL of dry THF. The mixture was stirred at 50 °C for 16 h. The solvent was then evaporated and the crude was purified by column chromatography (CH_2Cl_2/EtOAc) isolating the product. Yield: 0.59 g, 36%. ESI MS m/z ($C_{112}H_{178}Cr_7F_8N_3NiO_{36}$, M.W. = 2717.29 g mol^{-1}): 2739.7 $[M+Na]^+$. Elemental Analysis: Calculated (found) for $C_{112}H_{178}Cr_7F_8N_3NiO_{36}$: C, 49.51 (49.80); H, 6.60 (6.64); N, 1.55 (1.75); Cr, 13.39 (12.60); Ni, 2.16 (2.15).

1.4 Synthesis of $[Cr_7NiF_3(Etglu)(O_2C^{t}Bu)_{15}(OH_2)]$ **2.H₂O**:

2 was prepared following previous reported procedures.[18]

1.5 Synthesis of [(**2**){Cr_7NiF_3(Etglu)(O_2C^tBu)$_{15}$}$_2$] **5**:

2 (0.275 g, 0.1 mmol) and **3** (0.456 g, 0.2 mmol) were dissolved in warm acetone (100 mL) and stirred for 5 minutes. The precipitate was filtered and washed with acetone (100 mL). Then dissolved in 25 mL of pentane and filtered. To the solution of pentane, 10 mL of acetone was added and left undisturbed. Needle like crystals suitable for single crystal X-ray analysis were grown within two weeks. Yield: 0.1 g, 17%. Elemental Analysis: Calculated (Found) for $C_{278}H_{476}Cr_{21}F_{14}N_5Ni_3O_{106}$: C, 46.90 (47.88); H, 6.74 (6.99); N, 0.98 (0.93); Cr, 15.34 (13.35); Ni, 2.47 (2.28).

1.6 Synthesis of [$(CH_3CH_2CH_2)_2NH_2$][$Cr_7NiF_8(O_2C^tBu)_{16}$] **1**:

5 was prepared by following previous reported procedures.[28]

1.7 Synthesis of [$(CH_3CH_2CH_2)_2NH_2$][$Cr_7NiF_8(O_2C^tBu)_{14}(C_6H_5NO_2)_2$] **6**:

6 was prepared by following previous reported procedures.[29]

1.8 Synthesis of [**6**(Cr_7NiF_3(Etglu)(O_2C^tBu)$_{14}$)$_2$] **7**:

6 (0.200 g, 0.08 mmol) and **3** (0.377 g, 0.17 mmol) were dissolved in warm acetone for 5 minutes. The mixture was then filtered and the precipitate dissolved in pentane (50 mL). To the pentane solution, acetone (100 mL) was added and left undisturbed. Needle like crystals suitable for single crystal X-ray analysis were grown within one week. Yield: 0.12 g, 23 %. Elemental Analysis: Calculated (found) for $C_{254}H_{448}Cr_{21}F_{14}N_5Ni_3O_{102}$: C, 45.28 (45.13); H, 6.70 (6.66); N, 1.04 (1.13); Cr, 16.20 (15.43); Ni, 2.61 (2.67).

2. Thermogravimetric analysis (TGA)

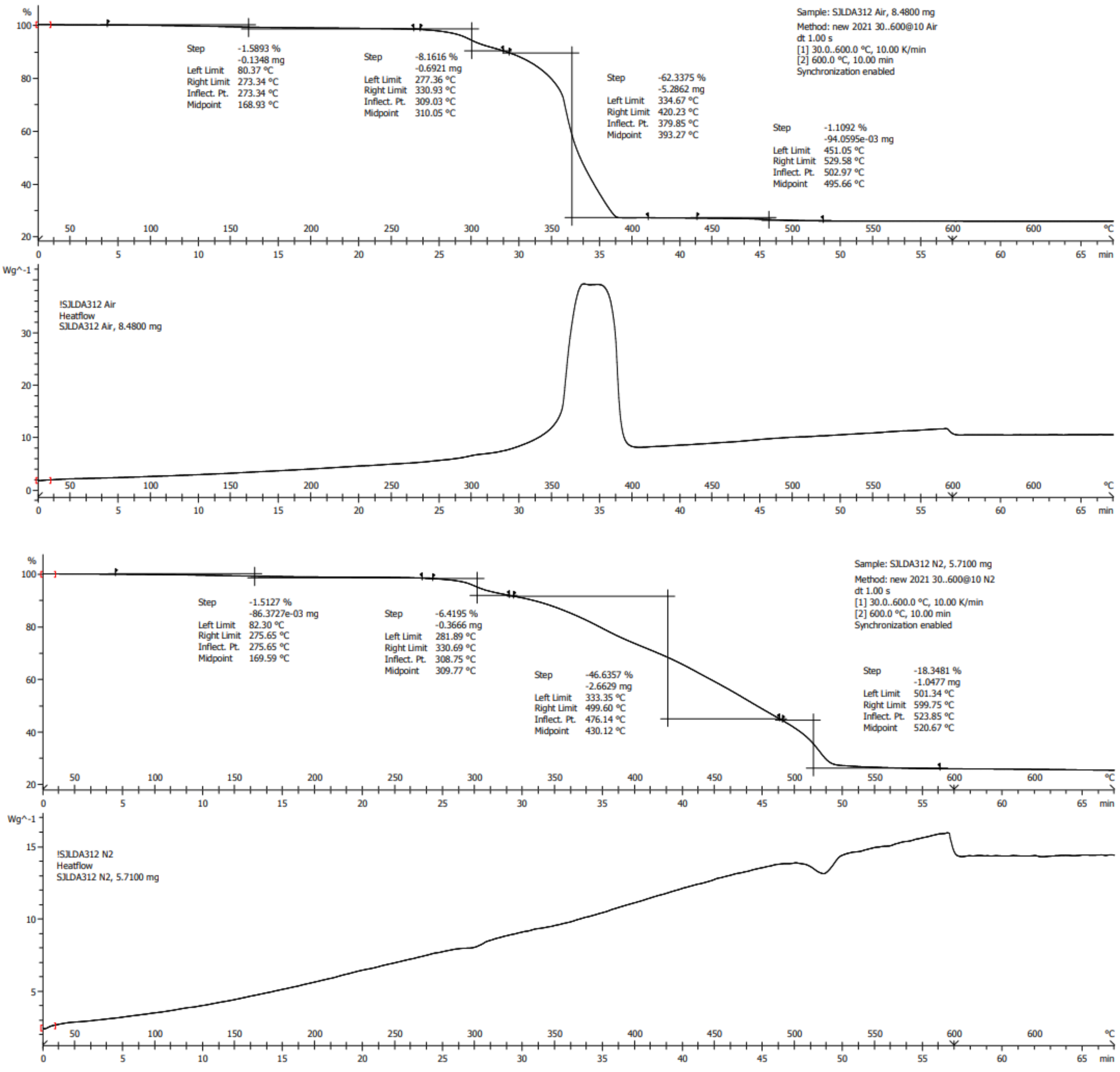

Figure S1: TGA for **5** under an atmosphere of air (top), and N_2 (bottom).

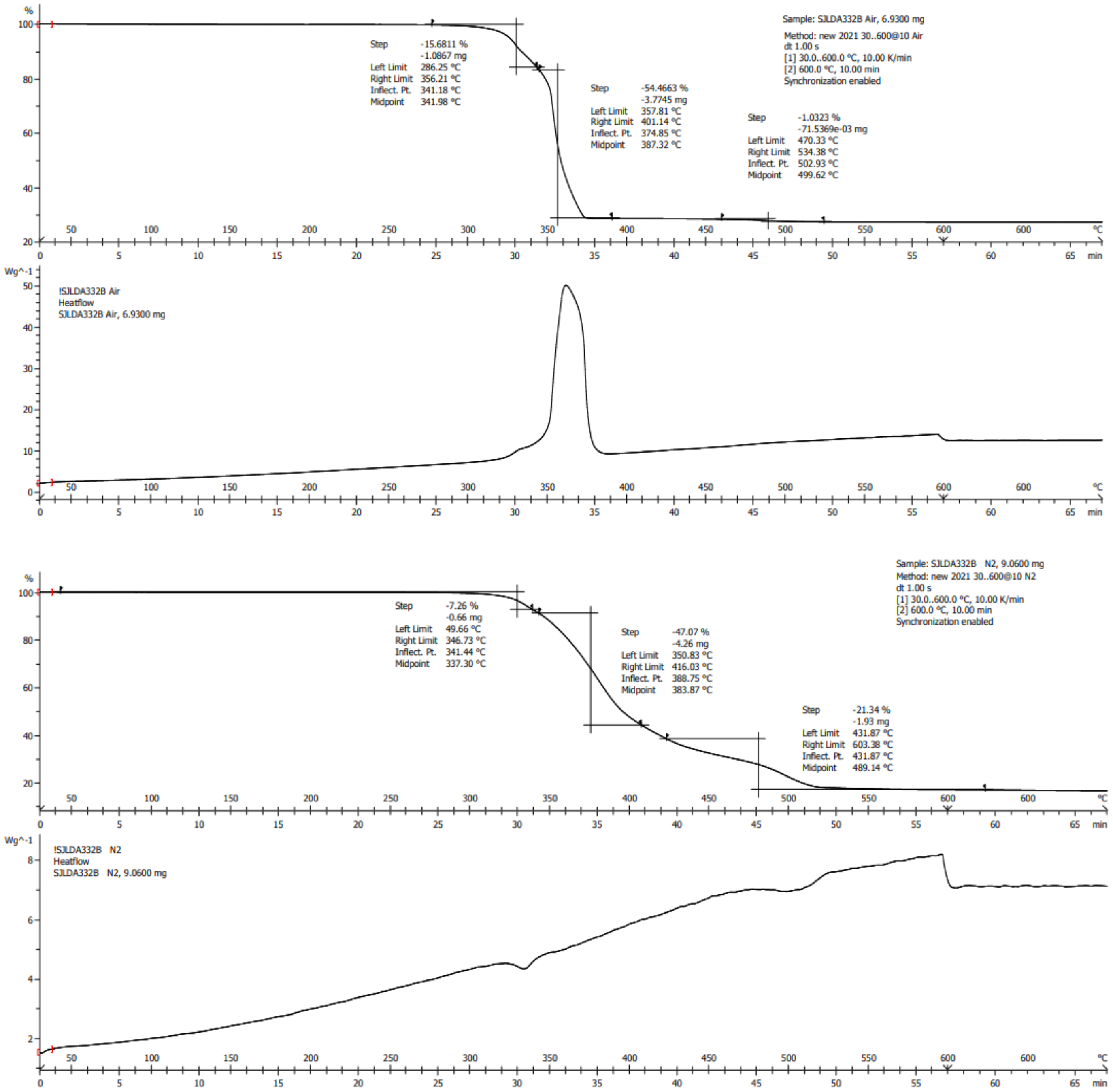

Figure S2: TGA for **7** under an atmosphere of air (top), and N_2 (bottom).

3. Crystallography

3.1 Crystallography Figures:

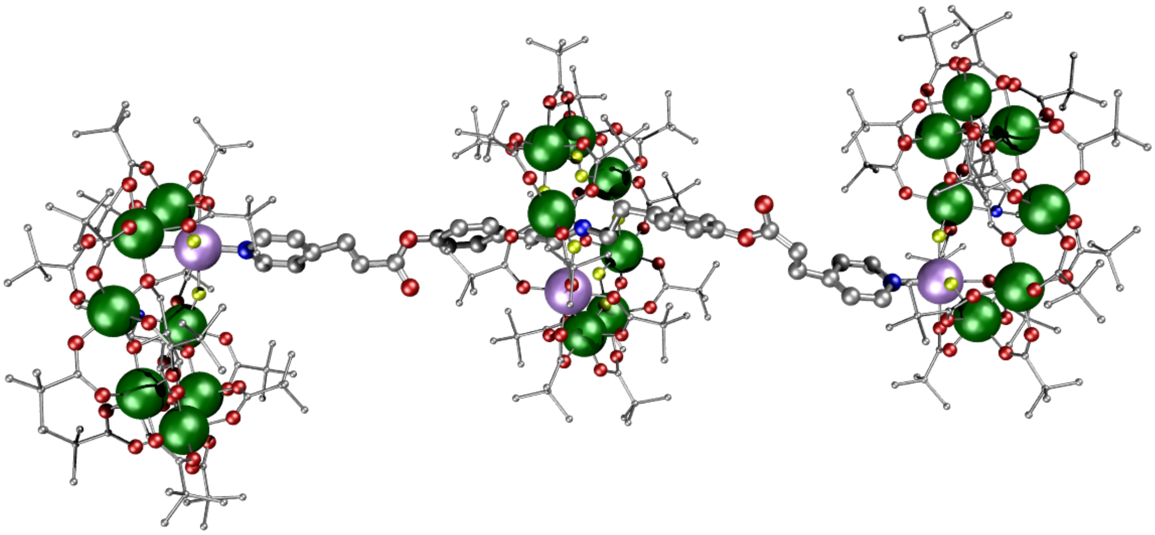

Figure S3: Crystal structure of **5**. Atom colours: Cr (green), Ni (lilac), O (red), N (blue), F (yellow), C (silver). Hydrogens omitted for clarity.

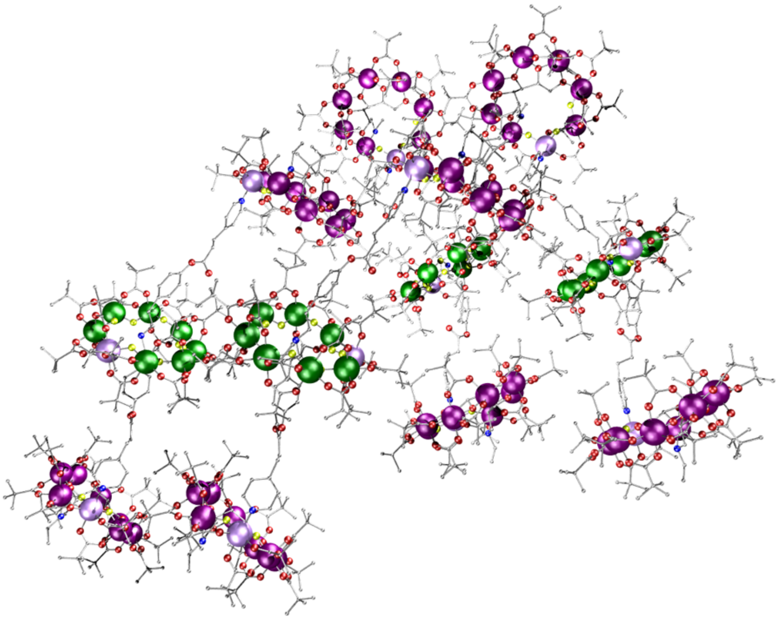

Figure S4: Packing diagram from crystal structure of **5**. Atom colours: Ni (lilac), O (red), N (blue), F (yellow), C (silver). Hydrogens omitted for clarity. Cr atoms labelled as green or purple to distinguish green and purple $\{Cr_7Ni\}$ rings, respectively.

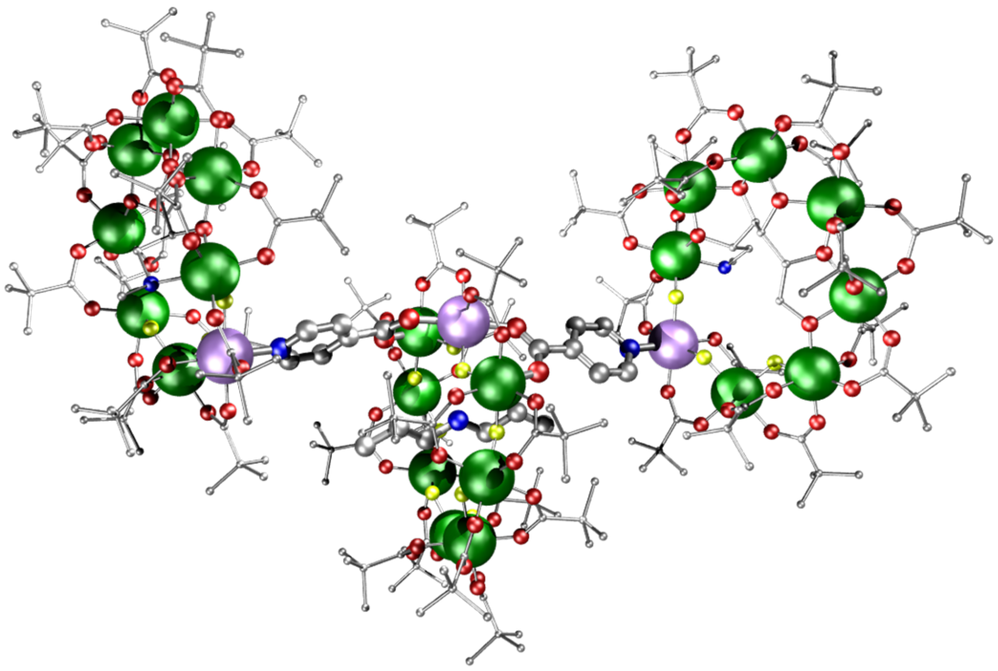

Figure S5: Crystal structure of **7**. Atom colours: Cr (green), Ni (lilac), O (red), N (blue), F (yellow), C (silver). Hydrogens omitted for clarity.

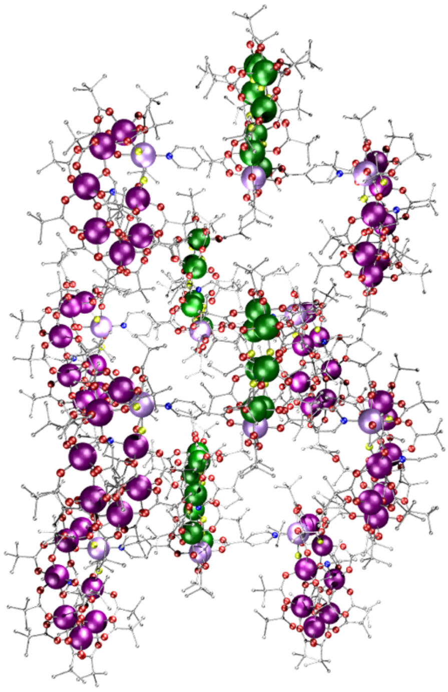

Figure S6: Packing diagram from crystal structure of **7**. Atom colours: Ni (lilac), O (red), N (blue), F (yellow), C (silver). Hydrogens omitted for clarity. Cr atoms labelled as green or purple to distinguish green and purple $\{Cr_7Ni\}$ rings, respectively.

3.2 Crystallographic Information:

X-ray data for compounds **3**, **5** and **7** were collected at a temperature of 100 K using a Rigaku FR-X Cu-Kα radiation equipped with a Hypix 6000HE detector, equipped with an Oxford Cryosystems nitrogen flow gas system. Data was measured using CrysAlisPro suite of programs. Absorption correction was performed using empirical methods (SCALE3 ABSPACK) based upon symmetry-equivalent reflections combined with measurements at different azimuthal angles.[31]

The crystal structure was solved and refined against all F^2 values using the SHELXL and Olex 2 suite of programmes.[32]

Table 1. Crystallography Data for **5** and **7**.

Compound	5	7
Empirical formula	$C_{278}H_{475}Cr_{21}F_{14}N_5Ni_3O_{107.5}$	$C_{278}H_{496}Cr_{21}F_{14}N_5Ni_3O_{110}$
M_w	5762.30 g mol^{-1}	7202.90 g mol^{-1}
Crystal System	Monoclinic	Monoclinic
Space Group	$P2_1$	$P2_1$
a (Å)	40.2789(8)	30.2894(6)
b (Å)	16.4456(3)	19.9198(4)
c (Å)	62.1465(17)	34.1717(7)
α	90	90
β	99.132(2)	102.257(2)
γ	90	90
V (Å^3)	40644.7(16)	20147.8(7)
Z	4	2
ρ_{calc} (g cm^{-3})	1.167	1.187
λ (Å)	1.54184	1.54184
T (K)	100.0(4)	240.0(4)
Goof on F^2	1.024	0.996
R indexes [I>=2σ (I)]	R_1 = 0.0955, wR_2 = 0.2318	R_1 = 0.0413, wR_2 = 0.0921
R indexes [all data]	R_1 = 0.1389, wR_2 = 0.2615	R_1 = 0.0599, wR_2 = 0.0989

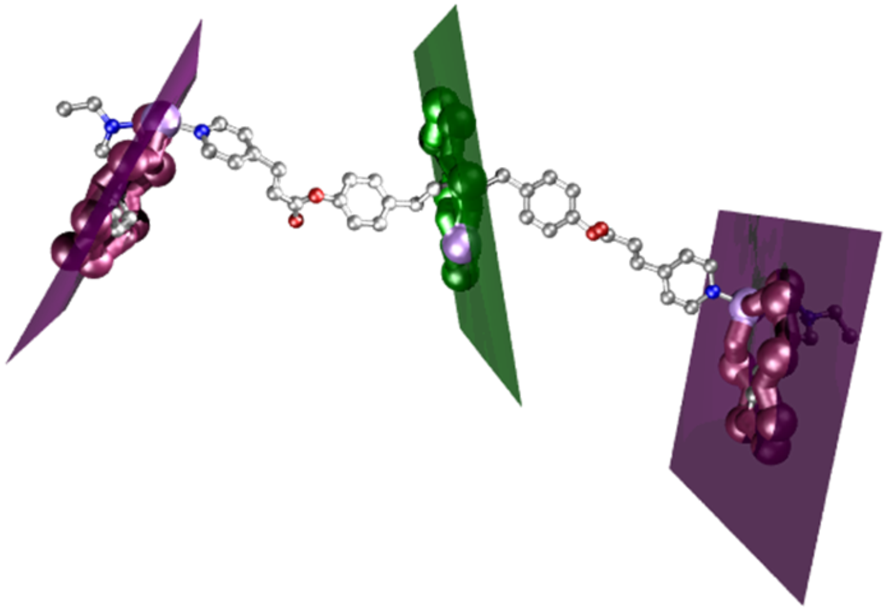

Figure S7: Dihedral angles between the ring planes for **5**: 37.35, 12.17 and 26.27 degrees, left ring - middle ring, middle ring – right ring, left ring – right ring, respectively.

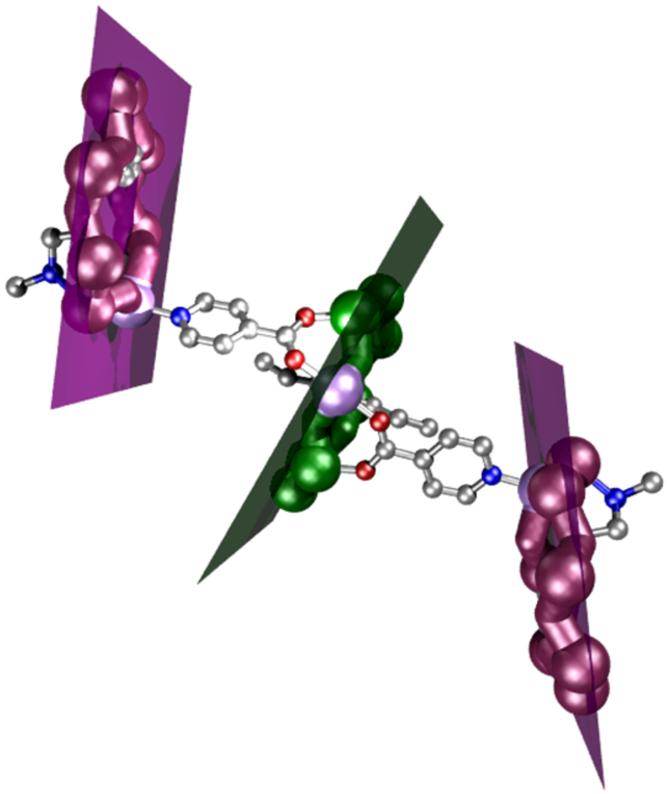

Figure S8: Dihedral angles between the ring planes for **7**: 17.59, 26.31 and 8.37 degrees, left ring - middle ring, middle ring – right ring, left ring – right ring, respectively.

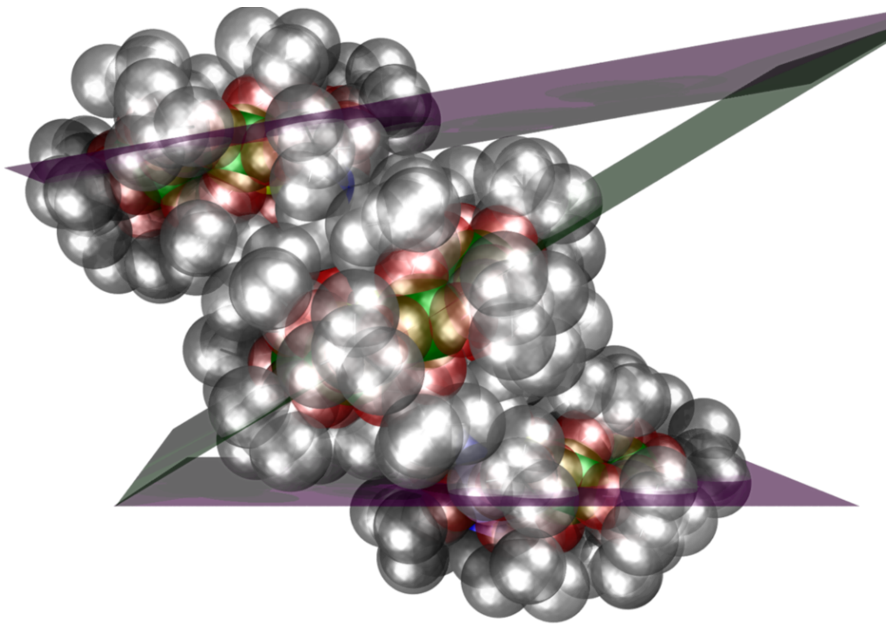

Figure S9: Space filling model with ring planes for **7**. Atom colours as per Figure S3.

4. Electron Paramagnetic Resonance:

Continuous wave Q-band (~34 GHz), K-band (~23 GHz) and X-band (~9.5 GHz) EPR spectra were recorded with a Bruker EMXPlus spectrometer equipped with a Bruker ER5106QT flexline resonator. Cryogenic temperatures were achieved using a Bruker Stringer closed cycle helium cryocooler mated to an Oxford Instruments CF935 cryostat. Temperature holding and regulation was controlled using an Oxford Instruments MercuryITC. The continuous wave data were collected on polycrystalline powders at 5 K (unless otherwise stated). All continuous wave spectra were field corrected using a Bruker 'Strong Pitch' standard (g = 2.0028) and all powder samples were checked for any polycrystalline nature, by measuring multiple random rotations.

Pulsed Q-band (~34 GHz) EPR data was collected on a Bruker ELEXSYS 580 FT spectrometer. The pulse data was collected from dry and degassed toluene solutions (0.2 mM) at 3 K (unless otherwise stated) using a Cryogenic cryogen free variable temperature cryostat incorporating a closed helium circuit. Simulations used EasySpin;[24] in order to incorporate the intrinsically broader linewidth of the green rings a g-strain of 1-2% was included for these components.

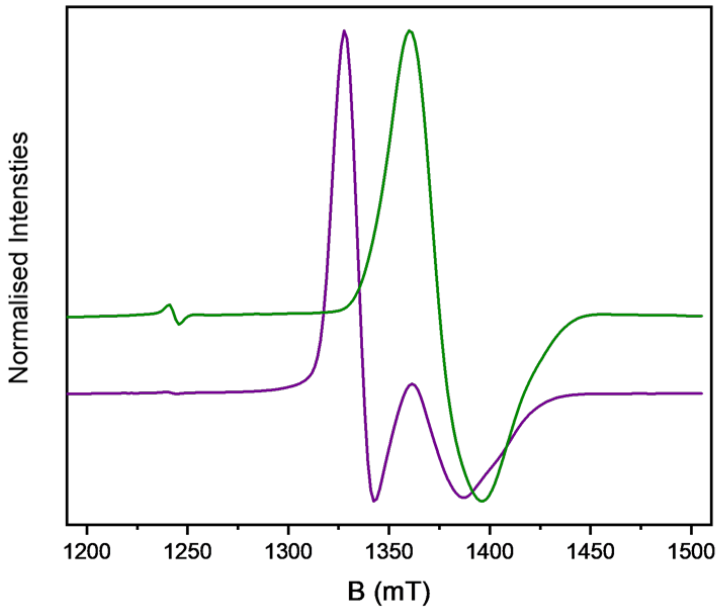

Figure S10: CW Q-band powder experiment at 5 K for **4** (green) and **5** (purple). Experimental frequency 34.024598 GHz.

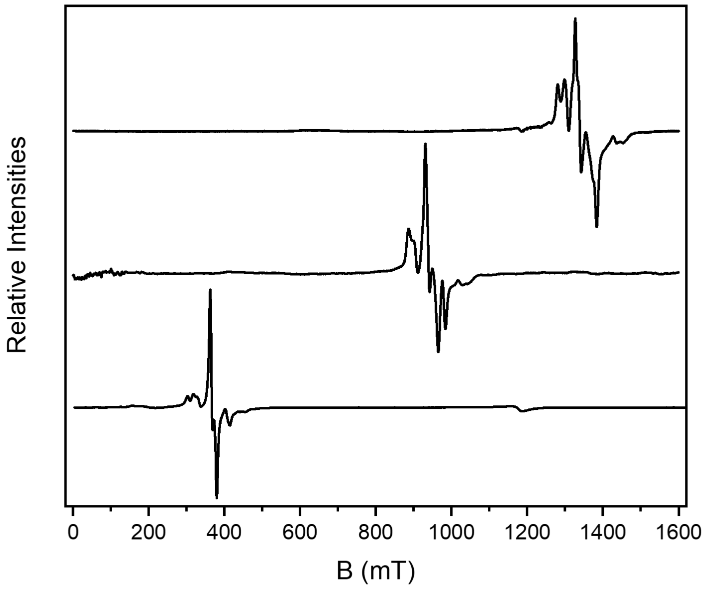

Figure S11: Full width spectra for **7** as a powder at 5 K, for Q-band (top), K-band (middle) and X-band (bottom). Experimental frequencies 34.018630, 24.055442 and 9.378597 GHz respectively.

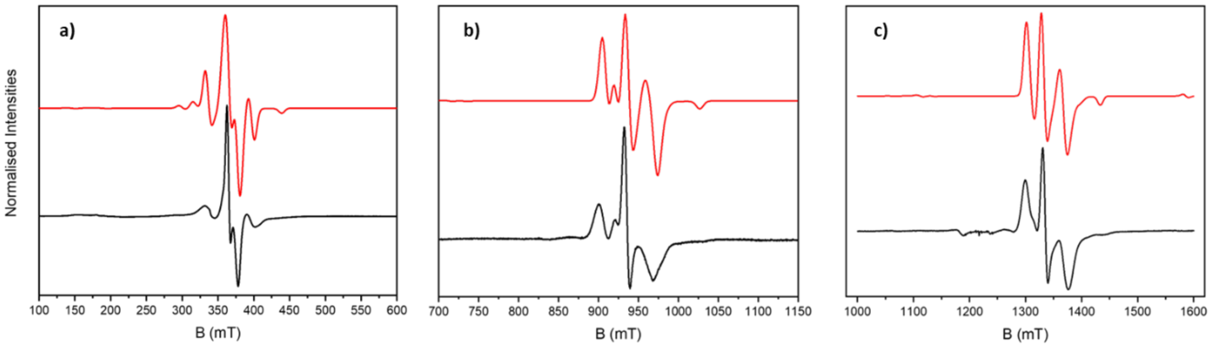

Figure S12: Experimental (black) and simulation (red) for **7**, as a 3 mM solution in 1:1 DCM and toluene at 5 K. Experimental frequencies for X-band: 9.367140 GHz (a), K-band: 23.987004 GHz (b) and Q-band: 34.097155 GHz (c). Simulation details as per manuscript, with no distribution of 'D^{ex}' parameter.

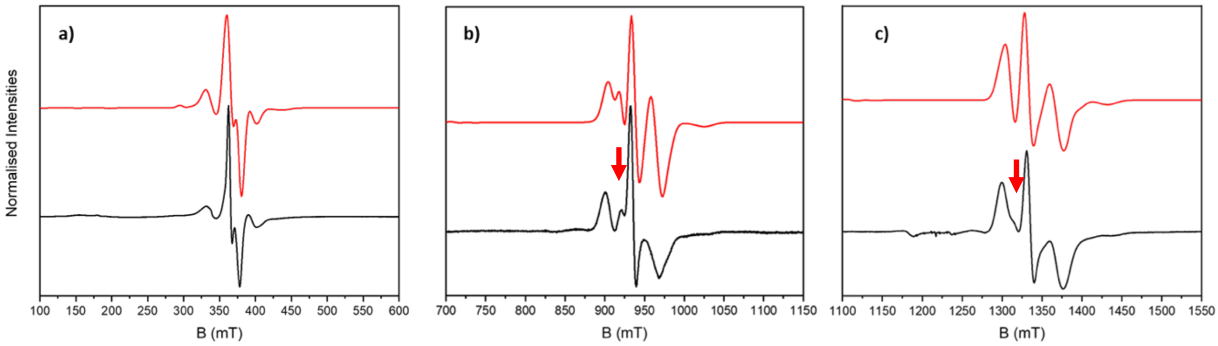

Figure S13: Experimental (black) and simulation (red) for **7**, as a 3 mM solution in 1:1 DCM and toluene at 5 K. Experimental frequencies for X-band: 9.367140 GHz (a), K-band: 23.987004 GHz (b) and Q-band: 34.097155 GHz (c). Simulation details as per manuscript, with distribution of 'D^{ex}' parameter. Red arrows mark resolved transitions within the |1,1/2> spin doublet state.

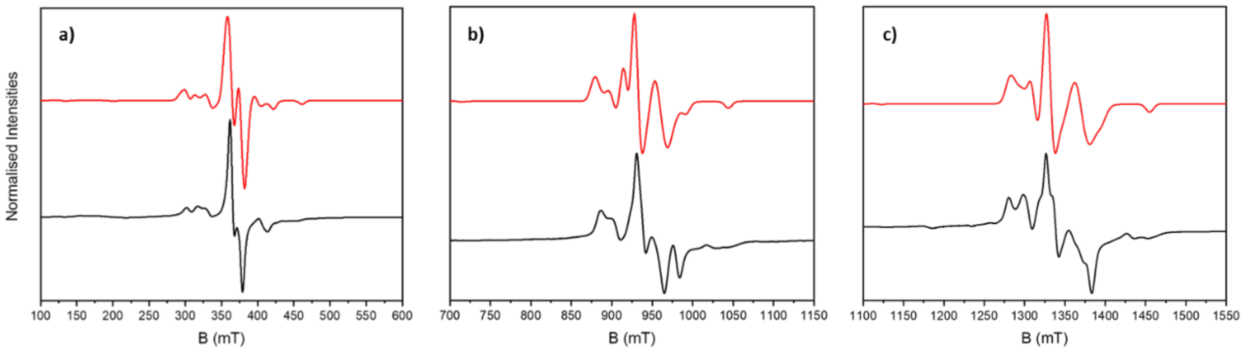

Figure S14: Experimental (black) and simulation (red) for **7**, as a powder at 5 K. Experimental frequencies for X-band: 9.378635 GHz (a), K-band: 23.872031 GHz (b) and Q-band: 34.098139 GHz (c). Simulation details as per manuscript, with no distribution of 'D^{ex}' parameter.

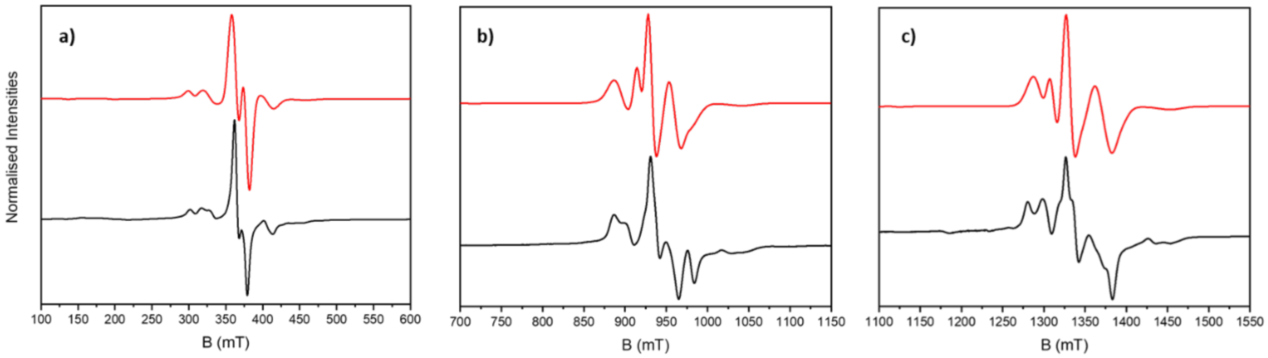

Figure S15: Experimental (black) and simulation (red) for **7**, as a powder at 5 K. Experimental frequencies for X-band: 9.378635 GHz (a), K-band: 23.872031 GHz (b) and Q-band: 34.098139 GHz (c). Simulation details as per manuscript, with distribution of 'D^{ex}' parameter.
